# Cerebellopontine epidermoid presenting with trigeminal neuralgia for 10 years: a case report

**DOI:** 10.1186/1757-1626-2-9345

**Published:** 2009-12-18

**Authors:** Yam B Roka, Prakash Bista, Gopal R Sharma, Pawan K Sultania

**Affiliations:** 1 Neurological Surgery Unit, Department of Surgery, B.P.Koirala Institute of Health Sciences, Dharan, 7053, Nepal; 2 National Neurosurgical Referral Centre, National Academy of Medical Sciences, Bir Hospital, Kathmandu, 13606, Nepal

## Abstract

Trigeminal neuralgia, also called tic douloureux, is a common and potentially disabling pain syndrome, which affects the trigeminal or fifth cranial nerve. The precise pathophysiology of Trigeminal neuralgia remains obscure. The disorder causes extreme, sporadic, sudden burning or shock-like face pain that lasts from few seconds to minutes and can be physically and mentally incapacitating. More than one nerve branch can be affected by the disorder. A 55-year-old female presented with pain over the left side of face for 10 years uncontrolled with carbamazepine. On examination the positive findings were reduced sensation by 25% over the left side of face with House and Brackman grade II facial nerve palsy. The corneal reflex was absent on left side. Magnetic resonance imaging showed left cerebellopontine angle (CPA) mass suggestive of an epidermoid involving the Vth nerve and Gasserian ganglion and extending into the middle cranial fossa. She underwent left suboccipital craniectomy and near total excision of the tumor with decompression of the V^th ^nerve which was fully engulfed by the tumor. Postoperative the VII nerve palsy increased to grade III and she had 50% loss of sensation over left side. She had no further attacks of pain and hence tapered off the carbamazepine. TN caused by cerebellopontine angle epidermoids is uncommon and should be kept in view in all cases presenting with TN. The aim of surgery for epidermoids is to decompress the cranial nerves and brain stem and not total removal with its attendant morbidity and mortality.

## Background

Trigeminal neuralgia (TN), also called tic douloureux, is a common and potentially disabling pain syndrome, which affects the trigeminal or fifth cranial nerve. The precise pathophysiology of TN remains obscure. The most common cause for TN is compression of the fifth nerve at its root entry zone by a vascular loop which was first reported by Janetta in 1957 [1]. The disorder causes extreme, sporadic, sudden burning or shock-like face pain that lasts from few seconds to minutes and can be physically and mentally incapacitating. More than one nerve branch can be affected by the disorder. There are no specific tests to diagnose TN, the diagnosis of which is mainly done clinically and with help of MRI/MRA. The House and Brackmann score helps in checking the progress or improvement of symptoms with or without surgical intervention [2]. Intracranial epidermoids are slow growing benign tumors which contain keratin, cellular debris and cholesterol, and lined with stratified squamous epithelium. These tumors comprise 1% of all intracranial tumors and most of them are located in the CP angle, suprasellar and third ventricle. Due to the rarity of epidermoids presenting as TN, most of the cases reported are limited to case reports or short series. We present such a case of TN secondary to CP angle epidermoid in a 55 year old lady.

## Case presentation

A 55-year-old Nepalese female presented with pain over left side of face for 10 years uncontrolled with carbamazepine. The pain was sudden, severe, lasting for few seconds, and aggravated by taking food. She had mild numbness over the left side. She also had drooling of saliva on the same side for last few months. On examination the only positive findings were reduced sensation by 25% over the left half of the face with House and Brackman grade II facial nerve palsy. She had involvement of all the three divisions of the Vth cranial nerve. The corneal reflex was absent on left side.

**Figure 1:**
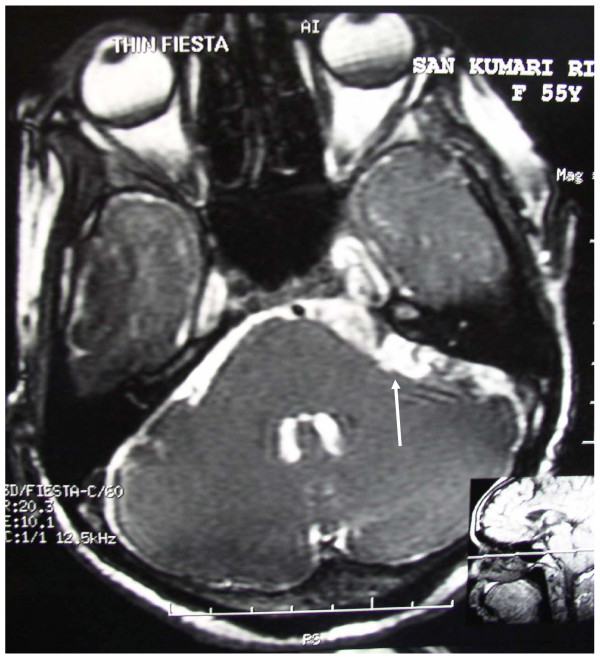
**MRI (FIESTA) showing the left CP angle epidermoid (arrow) involving the Vth nerve and Gasserian ganglion**.

MRI showed a left CP angle mass with features suggestive of an epidermoid involving the Vth nerve and Gasserian ganglion (Figure 1). She underwent left suboccipital craniectomy and near total excision of the tumor. Intraoperatively there was a pearly white capsulated mass with flakes arising from the CP angle and extending over the petrous bone into middle cranial fossa. Complete decompression of the Vth nerve, which was fully encased in the tumor, was achieved. There was no arterial loop over the root entry zone of the Vth nerve which usually is the most common cause for TN. The VIIth-VIIIth nerve complex, which was partially involved on its superior aspect, was also decompressed after mobilization of the anterior inferior cerebellar artery. Postoperatively the VII nerve palsy increased to grade III and she had 25% recovery of sensation over left side. She had no further attacks of pain and hence was tapered off carbamazepine.

## Conclusions

Intracranial epidermoids are slow growing benign tumors which contain keratin, cellular debris and cholesterol, and are lined with stratified squamous epithelium. These tumors comprise 1% of all intracranial tumors and most of them are located in the CP angle, suprasellar and third ventricle [3]. Due to the rarity of this presentation, most of the cases reported are limited to individual case reports or short series [4-7]. Meng et al., who reviewed 24 cases (one of the larger series), found that an epidermoid may be the cause of TN in younger patients [8]. In a series of 25 CP angle epidermoids, Mohanty et al. found TN due to the epidermoid in 13 cases with 7 of them presenting purely with TN. Eighteen of these 25 had supratentorial extension. The surgeons achieved complete excision in twelve patients, near total excision in eight, and partial excision in five cases [9].

Due to the proximity of CP angle epidermoids to the brainstem and cranial nerves, total excision is not possible in all cases. Unless the tumor is easily peeled off these structures, caution must be exercised to avoid an overzealous aim for total removal. Many studies and case reports support the view that total resection should be abandoned if the epidermoid is near the brainstem or cranial nerves [5,8,9]. Meng et al also suggested that in addition to complete resection of the tumour, arterial compression at the root entry zone of the trigeminal nerve should be sought, and if found, a microvascular decompression should be performed. Talacchi et al., in their series of 28 cases of posterior fossa epidermoids (with 20 of them in the CP angle), concluded that epidermoids localized to their primary origin site and those without supratentorial extension have the best chance of complete removal [10]. Malignant transformation into carcinoma, although rare, has also been reported in the literature [11]. Some of the other common presenting symptoms of CP angle epidermoids are hemifacial spasm and tinnitus [12].

Especially in the young, TN may be a manifestation of an epidermoid. In all cases of TN, an aggressive search must be made for the cause bearing in mind that vascular compression of the nerve may not be the only cause for TN. Epidermoid should always be included in the differential and sought with relevant investigations. The widely accepted House Brackmann scale is the best method to analyze the pre and post procedural improvement. The goal of surgery is decompression of the Vth nerve with relief of symptoms and complete excision should be avoided in cases of epidermoid adherent to cranial nerves or the brain stem.

## Consent

Written informed consent was obtained from the patient for publication of this case report and accompanying images. A copy of the written consent is available for review by the Editor-in-Chief of this journal.

## Competing interests

The authors declare that they have no competing interests.

## Authors' contributions

YBR, PB and GRS made substantial contributions to concept and design of the article and acquisition of materials. YBR and PK contributed significantly in critical revision and drafting the manuscript. All authors read and approved the final version of the manuscript.
